# Poly(oligo(ethylene glycol) methyl ether methacrylate) Capped pH-Responsive Poly(2-(diethylamino)ethyl methacrylate) Brushes Grafted on Mesoporous Silica Nanoparticles as Nanocarrier

**DOI:** 10.3390/polym13050823

**Published:** 2021-03-08

**Authors:** Khalid M. Alotaibi, Abdurrahman A. Almethen, Abeer M. Beagan, Latifah H. Alfhaid, Maqusood Ahamed, Ahmed M. El-Toni, Abdullah M. Alswieleh

**Affiliations:** 1 Department of Chemistry, College of Science, King Saud University, Riyadh 11451, Saudi Arabia; khalid.m@ksu.edu.sa (K.M.A.); abeagan@ksu.edu.sa (A.M.B.); 2 King Abdullah Institute for Nanotechnology, King Saud University, Riyadh 11451, Saudi Arabia; mahamed@ksu.edu.sa (M.A.); aamohammad@ksu.edu.sa (A.M.E.-T.); 3 King Abdulaziz City for Science and Technology, Riyadh 11451, Saudi Arabia; 4 Department of Physics, College of Science, University of Ha’il, Ha’il 2240, Saudi Arabia; l.alfhaid@uoh.edu.sa

**Keywords:** hybrid mesoporous silica nanoparticles, pH-responsive polymer brushes, surface modification, anti-cancer drug, drug delivery nanosystem

## Abstract

In this paper, a new pH-responsive nanosystem based on mesoporous silica nanoparticles (MSNs) was developed for cancer therapy. Poly(2-(diethylamino) ethyl methacrylate) (PDEAEMA) was grafted on their outer surface and acts as a gatekeeper, followed by subsequent modification of the polymer by cysteine (MSN-PDEAEMA-Cys) and poly(oligo(ethylene glycol) methyl ether methacrylate) (MSN-PDEAEMA-Cys-POEGMEMA). The physicochemical properties of these nanocarriers were characterized using scanning and transmission electron microscopies (SEM and TEM), Fourier-transform infrared spectroscopy (FTIR), X-ray photoelectron spectroscopy (XPS), and dynamic light scattering (DLS). The synthesized nanoparticles were well-dispersed with a diameter of ca. 200 nm. The obtained XPS results confirm the successful modification of MSN-PDEAEMA with Cys and POEGMEMA by increasing the peak intensity of C–O and C=O groups at 286.5 and 288.5 eV, respectively. An anti-cancer drug, doxorubicin (DOX), was encapsulated into the fabricated nanoplatform. The DOX release amount at physiological pH of 7.4 was limited (10%), while an accumulation drug release of ca. 35% was accomplished after 30 h in acidic media. The MTT cell line was used to assess the cytotoxicity of the unloaded and DOX-loaded fabricated nanoplatforms. Upon loading of DOX on these nanomaterials, they showed significant toxicity to human liver cancer cells. These results suggest that the prepared nano-structured materials showed good biocompatibility as well, and they can serve as nanocarriers for the delivery of anti-cancer drugs.

## 1. Introduction

Cancer is considered to be the second leading cause of death worldwide, accounting for an estimation of over 10 million deaths in 2020 [1]. Cancer can proliferate indefinitely and migrate easily to normal tissues. The excessive metabolite accumulation of the biological microenvironment in cancerous tissues is different from normal tissues, leading to acidic and hypoxic features [2,3]. Multi-sensitive drug delivery systems (DDSs) have been designed and developed to selectively release drug molecules in the microenvironment of diseased tissues and significantly enhance anti-cancer activity [4,5,6]. However, the psychological effects of complex environments, such as prolonged blood circulation, tumor tissue penetration, and cellular internalization, may hinder the drug-loaded nanocarriers from accessing the desired sites of tumor cells [7,8,9]. Thus, the ideal drug delivery system should have the ability to release the therapeutic agent in the right dose and at the right site within an acceptable period of time [10].

Mesoporous silica nanoparticles (MSNs) have attracted much research attention as drug carriers for their potential biomedical applications [11,12,13,14,15]. Silica is generally recognized as safe by the Food and Drug Administration (FDA) [11]. MSNs offer several unique properties and advantageous structures, such as high surface area and pore volume, uniform pore size, stable mesostructure [16], modifiable morphology [17], and excellent biocompatibility [18]. Despite the potential advantages of MSNs for drug delivery applications, pure MSNs tend to aggregate when directly exposed to biological environments, leading to uncontrolled drug release patterns [19,20]. These drawbacks could be minimized by using biocompatible polymers to coat the surface of silica nanoparticles in order to form different structures such as yolk–shell [21], core–shell [22], and brushes [23]. The combination of such polymers and silica nanoparticles not only addresses the aforementioned limitations but can also endow these particles with encapsulated guest molecules and subsequently release them at a later stage in an optimal way [24]. Polymeric nanostructures could remain stable in biological environments and minimize the side effects and the toxicity of DDSs, followed by a high-speed release rate of the drug in the diseased tissues [25,26,27,28,29]. Tincu et al. demonstrated successful encapsulation and controlled release of two hydrophobic drugs, natural curcumin (Cur) and synthetic 5-fluorouracil (5-FU), loaded in smart polymeric micelles (PMs) of a well-defined pH-sensitive poly(2-vinyl pyridine)-b-poly(ethylene oxide) (P2VP90-b-PEO398) block copolymer as nanocarriers [30].

Various stimuli-responsive drug-loaded systems have been developed during recent years [31,32,33,34]. pH-responsive polymers are one of the most utilized stimuli-responsive strategies for targeting tumor tissues due to the difference in the pH values between normal tissues and cancerous tissues (i.e., tumors (pH ~5.0)) [35]. The structure of pH-responsive polymers changes when pH values change. For instance, base polymers, such as poly(*N*,*N* diethylaminoethyl methacrylate), poly(*N*,*N*-dimethylaminoethyl methacrylate), and poly(b-amino ester), undergo structural changes when the amine groups become protonated. Protonation of the tertiary amino groups of poly(2-(diethylamino)ethyl methacrylate) (PDEAEMA) in an acidic medium triggered the swelling of the polymer due to the phase change from hydrophobic to hydrophilic with decreasing the environmental pH [22]. Alswieleh et al. successfully incorporated poly(2-(tert-butylamino)ethyl methacrylate) brushes into mesoporous silica nanoparticles via surface-initiated atom transfer radical polymerization (SI-ATRP). The results showed that the polymer brushes were highly swollen when they became protonated at a low pH value and collapsed when deprotonated at pH above 7.5 [23]. Beagan et al. modified the surface of hollow mesoporous silica nanoparticles with glucosamine-poly(2-(diethylamino) ethyl methacrylate) brushes. The acquired results showed an increase in the particle size from ca. 500 to ca. 980 nm when the pH decreased from 9 to 6.5, leading to the release of doxorubicin (DOX) from the nanosystem with a controlled manner [22]. Alswieleh et al. synthesized a diblock copolymer consisting of poly(ethylene glycol) methyl ether methacrylate and 2-(tert-butylamino)ethyl methacrylate grafted into the outer surface of MSNs via the atom transfer radical polymerization method. The nanoparticles exhibited a drug release efficiency of about 60% at pH < 6.5 [36]. More recently, the modification of PDEAEMA brushes’ surface with folic acid-coated magnetic nanoparticles was reported by Alswieleh and co-workers. The results suggested that DOX release from the fabricated nanosystem was ca. 20% in acidic media, compared to less than 5% in basic media [37].

The incorporation of poly(ethylene glycol) (PEG) into silica-based drug delivery systems can improve their therapeutic potential by enhancing the retention time of the therapeutics via increasing water solubility and protecting them from various enzymatic degradations inside a tissue or cell [38,39]. Yang et al. prepared a co-micellization of the diblock polymers poly(ethylene glycol)–PDEAEMA and poly(ethylene glycol) methyl ether-b-polycaprolactone (MPEG–PCL), which showed a well-controlled release rate ability for DOX [40]. However, the synthesized nanomaterial exhibits relatively low stability and slight leakage of DOX in neutral conditions as a result of the high value of critical micellar concentration (CMC). Feng et al. designed amphiphilic block copolymers of poly(*N*,*N*-diethylaminoethyl methacrylate) and poly(poly(ethylene glycol) methyl ether methacrylate) as a platform for paclitaxel delivery [41]. The results showed a pH-triggered release behavior of paclitaxel in the intracellular environment with pH 5, in which 55% of paclitaxel was released. Kang et al. prepared mixed-block polymeric micelles composed of poly(ethylene glycol)-block-poly(l-lactide) (PEG–PLLA) and poly(ethylene glycol)-block-poly(d-lactide) (PEG–PDLA). The micelles exhibited better performance than either of the polymers alone [42]. PEGylation of MSNs’ surface is used to improve their diffusion into tumor tissues and enhance colloidal stability. It was found that PEG-functionalized MSNs, compared to the non-modified surface, provide a significant improvement in drug diffusion and increase the penetration of fluorophore into the mucosal layer to reach epithelial cells [43]. Studies showed that protein corona formation on MSNs leads to opsonization, and this was overcome by coating them with PEG chains at a specific density and molecular weight. The non-coated MSNs, in contrast, presented negative zeta potentials as a result of forming protein corona on their surface [44]. A steric stabilization can be obtained after using PEGylation that prevents recognition by opsonins and decreases cleavage by proteases [6].

In this present work, a new pH-responsive system based on a polyelectrolyte polymer grafted into mesoporous silica nanoparticles (MSNs) was developed for biomedical applications. Poly(2-(diethylamino) ethyl methacrylate) (PDEAEMA) was grown on the outer surface of MSNs using surface-initiated activators regenerated by electron transfer atom transfer radical polymerization (SI-ARGET-ATRP). The surface of PDEAEMA brushes was modified with cysteine (Cys) and subsequently functionalized with molecules of poly(oligo(ethylene glycol) methyl ether methacrylate) (POEGMEMA) via the thiol-Michael addition click reaction. The anti-cancer drug DOX was used as a model drug and encapsulated into the prepared nanoplatforms. The properties of these nanocarriers were characterized using a variety of techniques such as dynamic light scattering (DLS), X-ray photoelectron spectroscopy (XPS), Fourier-transform infrared spectroscopy (FTIR), scanning electron microscopy (SEM), and transmission electron microscopy (TEM).

## 2. Materials and Methods

### 2.1. Materials

The water used in this study was deionized water with a purity of 15 MΩ-cm obtained using an Elga Pure Nanopore system (Lane End, United Kingdom). Hexadecyltrimethylammonium bromide (CTAB, 98%), ammonia aqueous (28 wt%), tetraethyl orthosilicate (TEOS, 98%), ethanol (99.8%), methanol (99.8%), (3-aminopropyl) triethoxysilane (APTES, >98%), pyridine (analytical grade), dimethylformamide (99.9%), poly(oligo(ethylene glycol) methyl ether methacrylate) (POEGMEMA), acetone, dichloromethane (DCM), 2,2′-Bipyridine (bipy, 99%), 2-(diethylamino)ethyl methacrylate (DEAEMA) (99%), triphenylphosphine (PPh3, 99%), and 2-bromo-2-methylpropionylbromide (BIBB, 98%) were obtained from Sigma-Aldrich (Taufkirchen, Germany). Cupric bromide (CuBr_2_, 98%) was purchased from BDH (British Drug Houses, London, UK). Triethylamine (TEA, 99%) and sodium azide (NaN_3_,99%) were obtained from Loba Chemie. *N*-hydroxysuccinimide (NHS), *N*-(3-dimethylaminopropyl)-*N*′-ethylcarbodiimide hydrochloride (EDC), succinic anhydride, and doxorubicin hydrochloride (DOX) were purchased from Tokyo Chemical Industry (Tokyo, Japan). Tetrahydrofuran (THF) was obtained from Nexgen Chemicals. l-ascorbic acid (98%) was obtained from Riedel-de Haën. Ammonium nitrate (NH_4_NO_3_, 99%) was purchased from Winlab (Harborough, UK). Potassium chloride, sodium chloride, sodium carbonate, potassium dihydrogen phosphate, and disodium hydrogen phosphate were purchased from Alfa Aesar (Lancashire, UK). All chemicals and reagents were utilized as supplied, without further purification.

### 2.2. Methods

#### 2.2.1. Synthesis of Mesoporous Silica Nanoparticles

Briefly, a solution containing 160 mL deionized water (160 mL), 1.0 g CTAB, and 7.0 mL concentrated ammonium hydroxide was placed in a 250 mL three-neck round-bottom flask and stirred until a clear solution was formed. Next, a mixture solution of TEOS (5 mL) and *N*-hexane (20 mL) were added dropwise into the above solution under continuous stirring. The solution was gradually turned to a milky slurry in 5 min under continuous stirring (200 rpm) at 35 °C. After further stirring for 10 h, silica nanoparticles were washed with ethanol and water by centrifugation. Finally, the collected sample was dried at 100 °C for two hours.

#### 2.2.2. The Modification of Mesoporous Silica Nanoparticles with an Amine Group (MSN–NH_2_)

Modification of the silica surface with amino group was performed as follows: 1 g of silica nanoparticles was dispersed in a solution containing 50 mL methanol and 0.5 mL APTES. The mixture was refluxed at 80 °C for 12 h. Finally, the particles were collected by centrifugation and washed several times with ethanol to remove the residual organics.

#### 2.2.3. Immobilization of BIBB Initiator on MSNs (MSN–Br) and Channel Formation

The synthesized MSN-NH_2_ (1 g) was dispersed in a solution containing 25 mL dichloromethane and 0.5 mL TEA (3.6 mmol). Next, a mixture containing 0.25 mL 2-bromo-2-methylpropionyl bromide (2.02 mmol) and 5 mL DCM was dropwise added to the suspension. The reaction was allowed to react under ambient temperature for 48 h. Finally, the modified silica nanoparticles (MSN–Br) were centrifuged and thoroughly washed with ethanol and DCM [19].

The organic surfactant was extracted by adding the initiated MSNs (1 g) into a solution containing ammonium nitrate (0.3 g) and ethanol (30 mL) at 75 °C overnight under stirring. The solid particles were collected and thoroughly washed with ethanol and then dried for 3 h at 60 °C.

#### 2.2.4. The Modification of MSNs with Poly(2-(diethylamino) ethyl methacrylate) Brushes (MSN–PDEAEMA)

MSN–PDEAEMA was prepared as follows: a mixture containing 0.4 g MSN–Br, 3 mL deionized (DI) water, and 12 mL ethanol was placed in a 25 mL round-bottom flask and degassed with nitrogen gas for 0.5 h under stirring. Then, 2-Diethylaminoethyl methacrylate (2 mL), 0.0009 g of CuBr_2_, and 0.0067 g of bipy were added to the mixture. Finally, ascorbic acid (7.6 mg) was added to the mixture under nitrogen and allowed to stir at ambient temperature for two hours. The solid particles were collected and thoroughly washed with an acidic aqueous solution followed by washing with ethanol and then dried in an oven at 60 °C.

#### 2.2.5. Coating the MSNs Surface with Amine Groups (MSN–PDEAEMA–NH_2_)

MSN–PDEAEMA (200 mg) was homogeneously dispersed in 10 mL of degassed DMF under nitrogen in a 25 mL round-bottom flask. A separated flask contained well-degassed solution of 0.2 M NaN_3_ in 5 mL DMF. The saturated solution of sodium azide was then added to MSN-PDEAEMA under N_2_ and the solution was heated for 18 h at 65 °C. Finally, the solid nanoparticles were collected by centrifugation and thoroughly washed with DMF.

The collected nanoparticles were then homogeneously dispersed in 10 mL of degassed DMF under nitrogen in a 25 mL round-bottom flask. A separated flask contained well-degassed solution of 0.2 M PPh_3_ in 5 mL DMF. The saturated solution of triphenylphosphine was then added to MSN–PDEAEMA under N_2_ and the solution was heated for 18 h at 65 °C. Finally, the solid nanoparticles were collected by centrifugation and thoroughly washed with DMF, water, and ethanol. The washed sample was added into a H_2_O/THF mixture and stirred under nitrogen atmosphere at 40 °C overnight. The solid nanoparticles were collected by centrifugation and thoroughly washed with ethanol.

#### 2.2.6. Immobilization of Cysteine on the Surface of MSNs (MSN–PDEAEMA–Cys)

To functionalize the nanoparticles with carboxylic groups, 100 mg of MSN-PDEAEMA–NH2 was added to a 10 mL solution of pyridine:DCM (1:1). Then, the solution was sonicated for 20 min. Briefly, 0.3 g of succinic anhydride was added to the reaction mixture and the solution was further sonicated for 0.5 h. After 18 h, the solid was centrifuged and washed with DMF.

The solid nanoparticles (100 mg) were dispersed in 10 mL DMF solvent under sonication for 20 min and then EDC (200 mg) and NHS (200 mg) were added to the mixture and sonicated for another 20 min. After 18 h, the nanoparticles were separated by centrifugation and washed with DMF.

To obtain MSN–PDEAEMA–Cys, 100 mg of MSN–PDEAEMA–NHS was added to DMF (10 mL) solution and placed in an ultrasonic bath for 25 min followed by the addition of cysteine (10 mg) and TEA (1 mL). The reaction mixture was then stirred for 18 h at room temperature. The nanomaterials were centrifuged and washed with DMF and DI water.

#### 2.2.7. Immobilization of POEGMEMA on the Surface of MSN–PDEAEMA (MSN–PDEAEMA-Cys-POEGMEMA)

To modify the nanoplatform with POEGMEMA, 100 mg of MSN-PDEAEMA-Cys was added to 20 mL solution of acetone:TEA (1:1) and sonicated for 10 min followed by the addition of 1 mL POEGMEMA and further sonication for 10 min. The reaction mixture was then stirred for 48 h at 25 °C. The sample was centrifuged and thoroughly washed with acetone, deionized water, and ethanol.

### 2.3. Measurement and Characterization

The mesostructure and the morphology of the nanoparticles were demonstrated using transmission electron microscopy (TEM, Tokyo, Japan). TEM images were obtained on a JEOL JEM-1400 microscope with an accelerating voltage of 100 kV. SEM images were obtained using a JEOL JSM-7610F microscope at 5 kV (Tokyo, Japan). Infrared (IR) spectra were recorded using potassium bromide pellets in the region of 4000–400 cm^−1^ with a resolution of 4 cm^−1^, using a PerkinElmer Spectrum BX FTIR spectrophotometer (Beaconsfield, UK). The X-ray photoelectron spectroscopy measurements were performed with a JEOL JPS-9030 Photoelectron Spectrometer 9 (Tokyo, Japan). Particle size was measured using DLS Malvern instruments (Zetasizer Nano ZS, Malvern, UK) at different pH values. UV/Vis spectra were recorded on a SpectraMax Plus 384 microplate reader. Thermogravimetric analysis (TGA) was conducted using a PerkinElmer Pyris instrument (Beaconsfield, IA, USA) with a temperature up to 800 °C at 10 °C/min heating rate.

### 2.4. Drug Loading and Release

The drug loading and release were conducted in accordance with the method of Bilalis et al. [45]. Briefly, 1 mg of the fabricated nanoparticles was suspended in 1 mL buffer solution followed by the addition of DOX solution (1 mL). Next, the solution’s pH was adjusted to 3 using 0.1 M hydrochloric acid and stirred for a day under dark conditions at room temperature. Then, the pH of the mixture was adjusted to 8 using aqueous solution of 0.1 M sodium hydroxide and stirred for another 2 h. Supernatants were taken gently after centrifugation to determine the concentration of unloaded drug by means of a UV–Vis spectrophotometer at wavelength of 480 nm.

The loading capacity (LC) and entrapment efficiency (EE) for DOX were calculated using the following Equations:

EE = (the amount of drug on the nanoparticles/initial amount of drug) × 100(1)

LC = [weight of DOX/(weight of nanoparticles + weight of DOX)] × 100(2)

The process of drug release was performed by adding 1 mg of DOX@MSN-PDEAEMA, DOX@MSN-PDEAEMA-Cys, or DOX@MSN-PDEAEMA-Cys-POEGMEMA into 1 mL phosphate-buffered saline (PBS) with different pH values, ranging from 5 to 8, under constant shaking at 37 °C. The solution was collected from the dispersion at different time intervals, and the amount of drug released was identified using UV–Vis spectroscopy. The volume of buffer was kept constant via the addition of fresh medium (1 mL) after each collection. The cumulative percent release of the drug was calculated using the formula given below:

LC = [weight of DOX/(weight of nanoparticles + weight of DOX)] × 100(3)

### 2.5. Cell Culture and Exposure Protocol

HepG2 cells (American Type Culture Collection (ATCC), Manassas, VA, USA) were cultured in Dulbecco’s Modified Eagle Medium (DMEM) (Invitrogen, Carlsbad, CA, USA) with 10% Fetal Bovine Serum (FBS) and antibiotics (100 µg/mL streptomycin + 100 U/mL penicillin). Cells were maintained in a humidified incubator under control conditions (37 °C and 5% CO_2_). Nanomaterials were suspended in culture medium (DMEM + 10% FBS) and diluted to the desired concentration (1–100 µg/mL). Different dilutions of nanoparticles were further sonicated utilizing a sonicator bath at room temperature for 10 min at 40 W to avoid agglomeration of nanoparticles before exposure to cells. Cells without nanoparticles served as control in each experiment.

#### MTT Cell Viability Assay

MTT assay [46] with some specific changes [47] was applied to examine the cytotoxicity of various types of MSNs. In brief, 20,000 cells/well were seeded in a 96-well plate and allowed to attach on the plate’s surface for 24 h. Then, cells were treated for 24 h with different concentrations of MSNs (1–100 µg/mL). After that, culture media were aspirated from each well and replaced with a new culture medium containing MTT solution. The plate was incubated for 3 h at 37 °C until a purple-color formazan product appeared. The formazan was dissolved in acidified isopropanol. Then, 100 µL solution was transferred to another 96-well plate. Absorbance of the solution was recorded at 570 nm with a microplate reader (Synergy-HT, BioTek, Winooski, VT, USA).

## 3. Results and Discussion

**Scheme 1:**
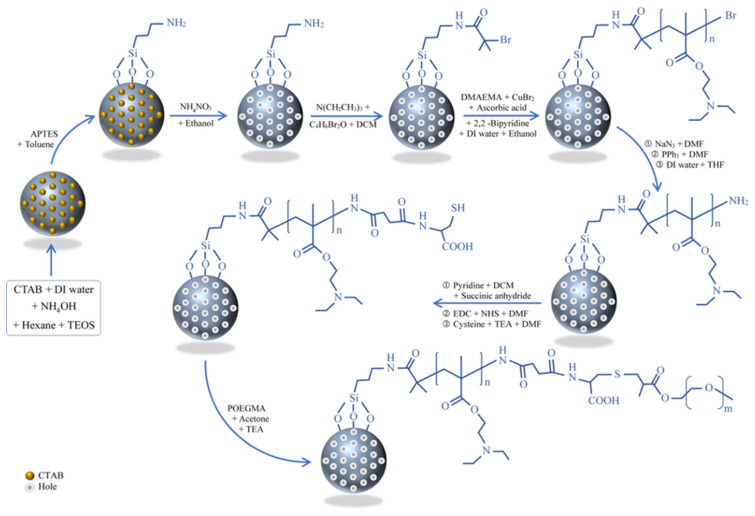
Synthesis procedure for poly(2-(diethylamino) ethyl methacrylate) (PDEAEMA) brushes capped with poly(oligo(ethylene glycol) methyl ether methacrylate) (POEGMEMA) on mesoporous silica nanoparticle (MSN) surfaces via surface-initiated activators regenerated by electron transfer atom transfer radical polymerization (SI-ARGET-ATRP).

As illustrated in Scheme 1, hybrid mesoporous silica nanoparticles were synthesized in multiple steps. APTES was conveniently attached to MSNs followed by the reaction of the amino groups with BIBB before the extraction of the organic surfactant (CTAB). CTAB was extracted using the solvent extraction method to keep the internal pore surface free to incubate guest molecules. PDEAEMA brushes were grown on the outer surface of MSNs via SI-ARGET-ATRP. The halogen atoms at the end of the PDEAMA chains were substituted to amines, using NaN_3_ and PPh_3_, followed by the reaction with succinic anhydride. NHS and EDC were utilized to activate the carboxylic groups to allow the reaction with amine groups in cysteine molecules. Then, POEGMEMA was used to cap the surface via the thiol-Michael addition click reaction in the presence of TEA.

**Figure 1:**
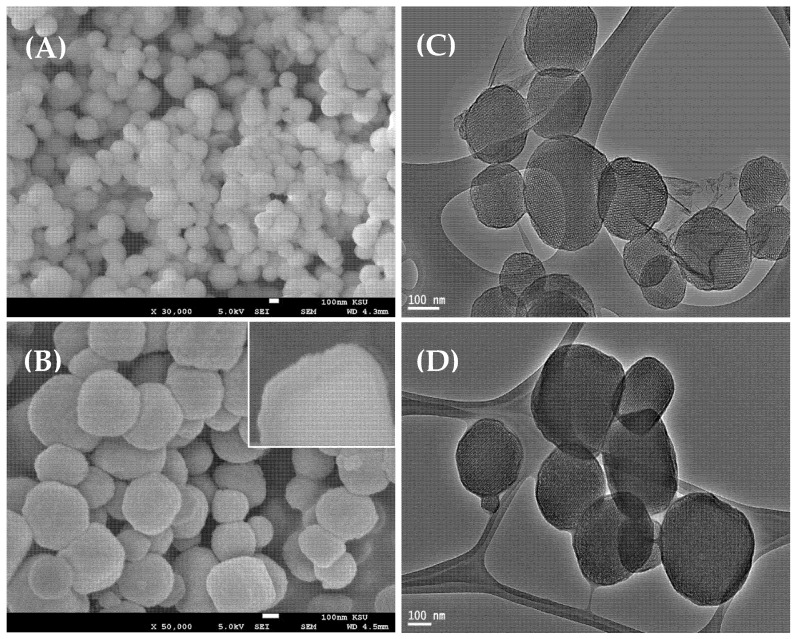
SEM and TEM images of fabricated mesoporous silica nanoparticles: (**A**) SEM image of unmodified MSNs, (**B**) SEM image of PDEAEMA-attached MSNs, (**C**) TEM image of as-made silica nanoparticles, and (**D**) TEM image of PDEAEMA-coated silica nanoparticles.

The size and shape of the fabricated nanoparticles were studied using scanning electron microscopy (SEM) and transmission electron microscopy (TEM). SEM images of the unmodified MSNs and MSN-PDEAEMA are shown in Figure 1. The first image shows that the as-made MSN samples were spherical, with an average diameter of ca. 220 nm (Figure 1A), whereas the average particle size was increased by 10% after the attachment of PDEAEMA to the surface of the MSNs, as shown in Figure 1B. TEM was also utilized for studying the morphology of the MSNs and MSN-PDEAEMA. As can be seen from the images, the as-made MSNs exhibit dispersed particles with an average particle size of 240 nm and a clear pore structure (Figure 1C). After being coated with PDEAEMA, a continuous layer, which illustrates an increase in darkness in compared to the bright inner core, can be clearly observed on the outer shell of the silica nanoparticles (Figure 1D). The dry thickness of this polymer layer was estimated to be ca. 15 nm.

**Figure 2:**
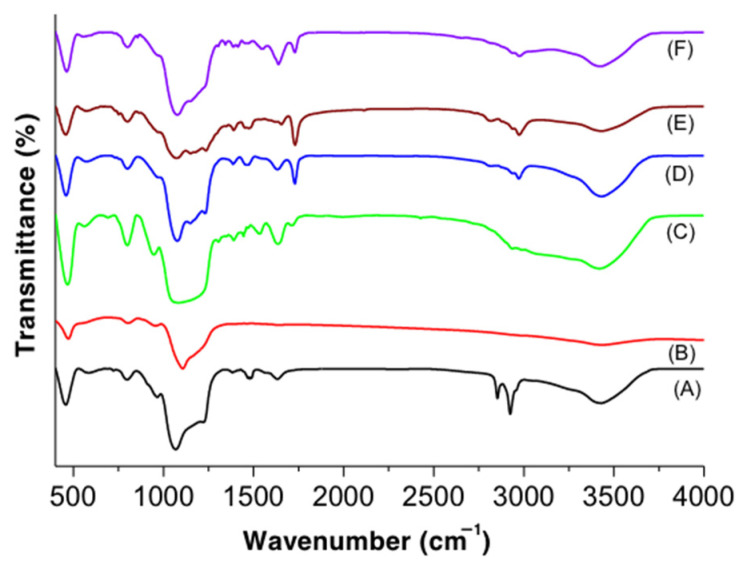
FTIR spectra of the modified silica nanoparticles: (**A**) as-made MSNs, (**B**) hexadecyltrimethylammonium bromide (CTAB)-free MSNs, (**C**) MSN–Br, (**D**) MSN-PDEAEMA, (**E**) MSN-PDEAEMA-cysteine (Cys), and (**F**) MSN-PDEAEMA-Cys-POEGMEMA.

FTIR spectra were obtained for as-made MSNs, CTAB-free MSNs, MSN-Br, MSN-PDEAEMA-Cys, and MSN-PDEAEMA-Cys-POEGMEMA to verify the molecules attached to the surface, as shown in Figure 2. A wide band at 1240–1030 cm^−1^ was observed, which was attributed to Si–O–Si band stretching of the condensed silica network, as well as a peak at 799 cm^−1^ for the stretching vibration of Si–O. The successful removal of the organic template was confirmed by the disappearance of the C–H stretching vibration at 2850 cm^−1^. After the initiation step, the C–H bending vibration of the methyl group was observed at 1450 and 1384 cm^−1^. A strong absorption peak at 1730 cm^−1^ was observed in all polymer-coated MSN samples which was associated with –C=O stretching vibrations, indicting the successful polymerization process.

**Figure 3:**
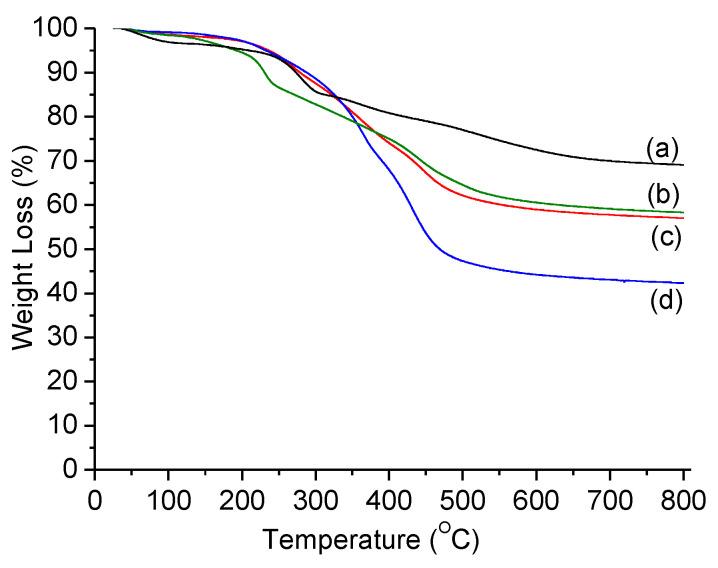
Thermogravimetric analysis (TGA) of the modified silica nanoparticles: (**a**) MSN-Br, (**b**) MSN-PDEAEMA, (**c**) MSN-PDEAEMA-Cys, and (**d**) MSN-PDEAEMA-Cys-POEGMEMA.

The surface investigations were also conducted using thermogravimetric analysis (TGA), as shown in Figure 3. The obtained results demonstrate that the weight loss of MSN-Br was ca. 25% at ~600 °C. After polymerization, the amount loss of organic layer was approximately 40%. The weight loss was ~45% when the surface of PDEAEMA brushes was modified with cysteine. A further reduction in weight was observed for MSN-PDEAEMA-Cys-POEGMEMA, indicating the successful attachment of POEGMEMA on the surface.

**Figure 4:**
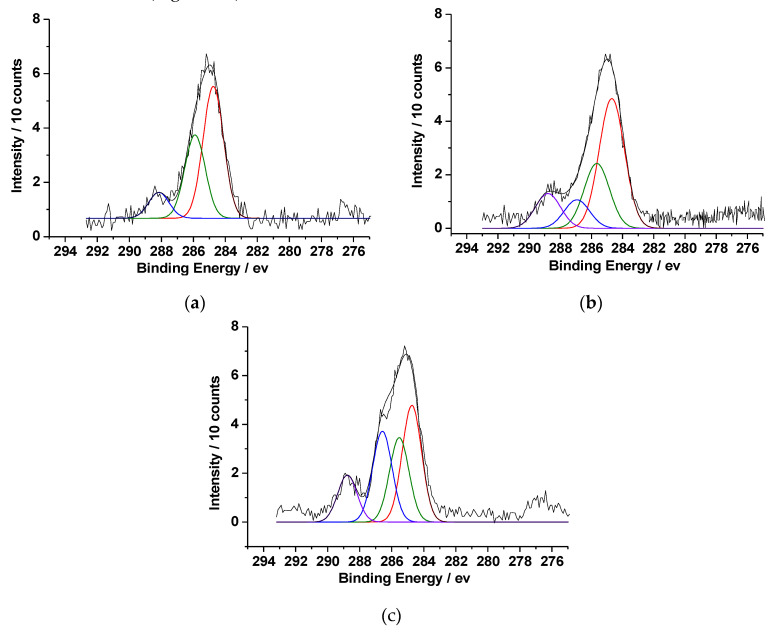
X-ray photoelectron spectra at the C1s region for (**a**) MSN-PDEAEMA, (**b**) MSN-PDEAEMA-Cys, and (**c**) MSN-PDEAEMA-Cys-POEGMEMA.

To confirm the successful reactions of Cys and POEGMEMA with the surface of the polymer brushes, XPS was used in the analysis of MSN-PDEAEMA, MSN-PDEAEMA-Cys, and MSN-PDEAEMA-Cys-POEGMEMA (Figure 4). Three components were fitted in the C1s spectra for MSN-PDEAEMA, as illustrated in Figure 4a. The binding energies of the fitted peaks were 284.9, 286, and 288.5 eV, which were assigned to C–H, C–*N*/C–O, and O=C, respectively. The peak area ratios between the three aforementioned components are similar to the theoretical peak area ratios of the polymer composition. It was observed that the peak intensity of C=O was increased for MSN-PDEAEMA-Cys due to the successful attachment of both succinic acid and cysteine on the polymer outer surface, as shown in Figure 4b. Due to the repeated C–O units in POEGMEMA molecules, the intensity of C–O peaks in the C1s region was expected to increase. As expected, there was an increase in the intensity of the peak at 286.5 eV after the immobilization of POEGMEMA, which confirms the successful attachment of these polymeric molecules (Figure 4c).

**Figure 5:**
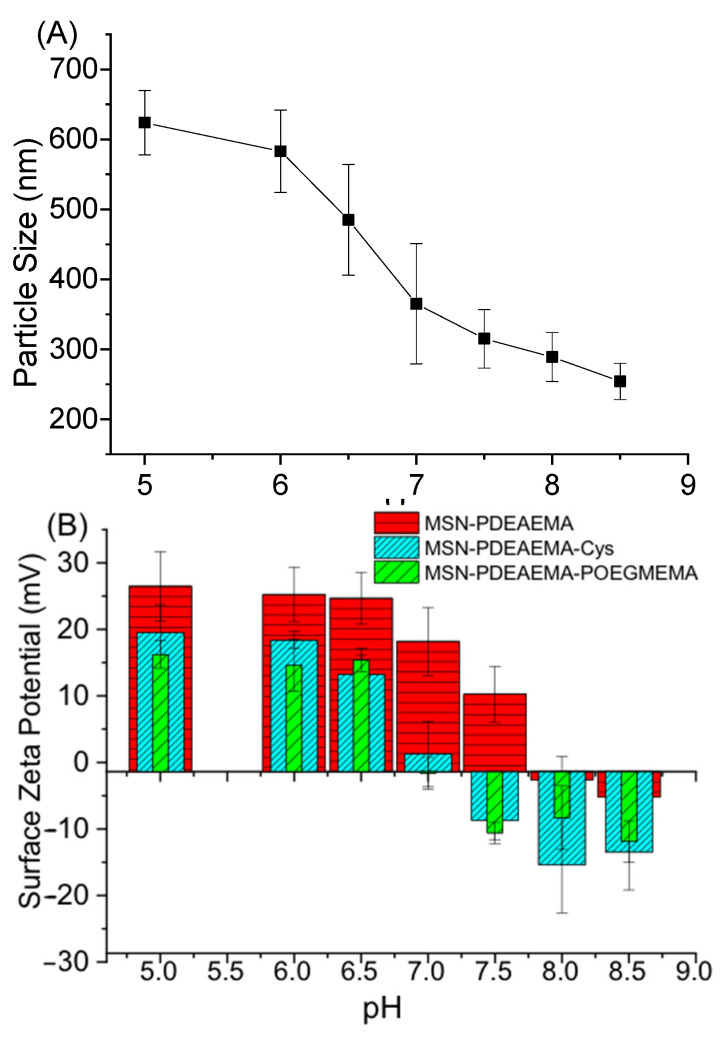
(**A**) The average particle size of MSN–PDEAEMA at different pH levels at 25 °C. (**B**) The surface zeta potential MSN-PDEAEMA, MSN-PDEAEMA-Cys, and MSN-PDEAEMA-Cys-POEGMEMA at different pH levels at 25 °C.

In order to examine the effect of the solution pH on the size of the designed nanocarriers, DLS measurements were performed in PBS at different pH values (Figure 5A). The results indicate that with decreasing the pH value, the size of MSN-PDEAEMA increased due to the protonation process of polymer amino groups. When the pH of the solution was increased, the particle size decreased, caused by the deprotonation of polymer amino groups. Figure 5B shows the zeta potential of the designed materials at different pH values. MSN–PDEAEMA exhibited a positively charged surface at pH below 7, with an average of 25 mV. At pH above 7.5, the surface charge was ca. −5 mV, resulting from the deprotonation process. However, MSN-PDEAEMA-Cys and MSN-PDEAEMA-Cys-POEGMEMA showed a negative surface charge with an average of −15 mV when the samples were exposed to a pH value above 7 due to the presence of carboxylic groups at the surfaces.

The drug-loading capacity of the MSN-PDEAEMA, MSN-PDEAEMA-Cys, and MSN-PDEAEMA-Cys-POEGMEMA nanosystems with DOX was found to be similar at ca. 69%. FTIR spectra confirmed the DOX loading into the nanocarriers via the clear appearance of two peaks at 1405 and 1576 cm^−1^ which were assigned to C–C stretches in the aromatic ring of the DOX molecule (Figure S1). Furthermore, a significant increase in the peak at 1620 cm^−1^ was observed, which is assigned to *N*–H stretching mode [48,49]. The average particle size and zeta potential of DOX-loaded MSN-PDEAEMA were found to be similar to unloaded DOX (Figure S2). DLS data indicate that the size of MSN-PDEAEMA increased with decreasing the pH value and reached ca. 600 nm at pH = 5. In basic medium, the particle size was decreased due to the deprotonation of polymer amino groups.

**Figure 6:**
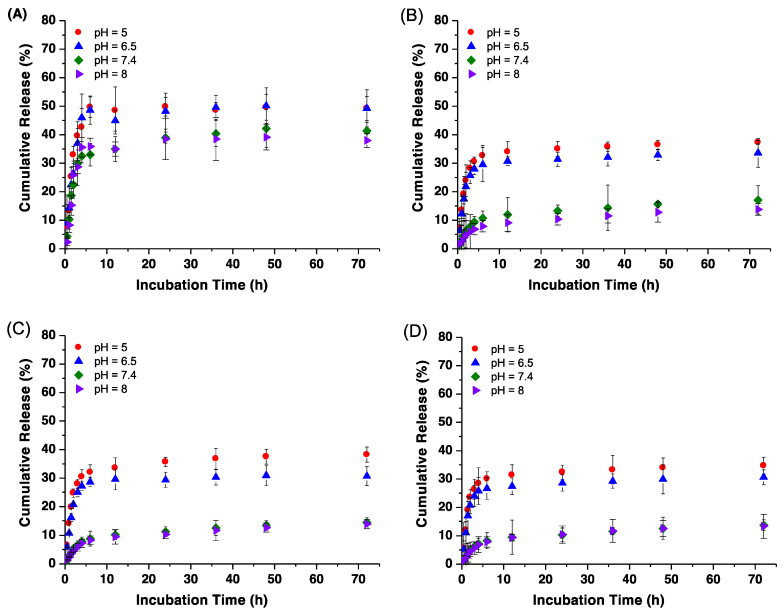
Drug release profiles of doxorubicin (DOX) loaded in (**A**) MSN-Br, (**B**) MSN-PDEAEMA, (**C**) MSN-PDEAEMA-Cys, and (**D**) MSN-PDEAEMA-Cys-POEGMEMA nanocarriers with different pH values at 37 °C.

The DOX release from these prepared nanocarriers was assessed at pH = 5, 6.5, 7.4, and 8 (PBS buffer). As shown in Figure 6, the drug release rate for all fabricated nanosystems was faster in mild acidic solutions compared to basic media. The highest cumulative drug release rate was observed when the particles were exposed to the solution with pH = 5. In acidic media, almost 50% of the loaded DOX was released from initiated nanoparticles within the initial 4 h and the accelerated drug release from these nanocarriers was ca. 55% after 8 h. In basic media, DOX was released from MSN–Br with an accelerated drug release of ca. 35% after 10 h due to the deprotonation process of DOX. However, approximately 30% of the loaded DOX was released from polymer-coated nanoparticles within the initial 5 h. After 10 h, the accelerated drug release from these nanocarriers was ca. 35%. This fast drug release was attributed to the protonation of the amino group present in the PDEAEMA, which resulted in an accelerated drug release. There was no significant difference in the DOX release profiles for all three nanosystems, indicating that the cysteine and POEGMEMA molecules that capped MSN-PDEAEMA did not affect the drug release.

**Figure 7:**
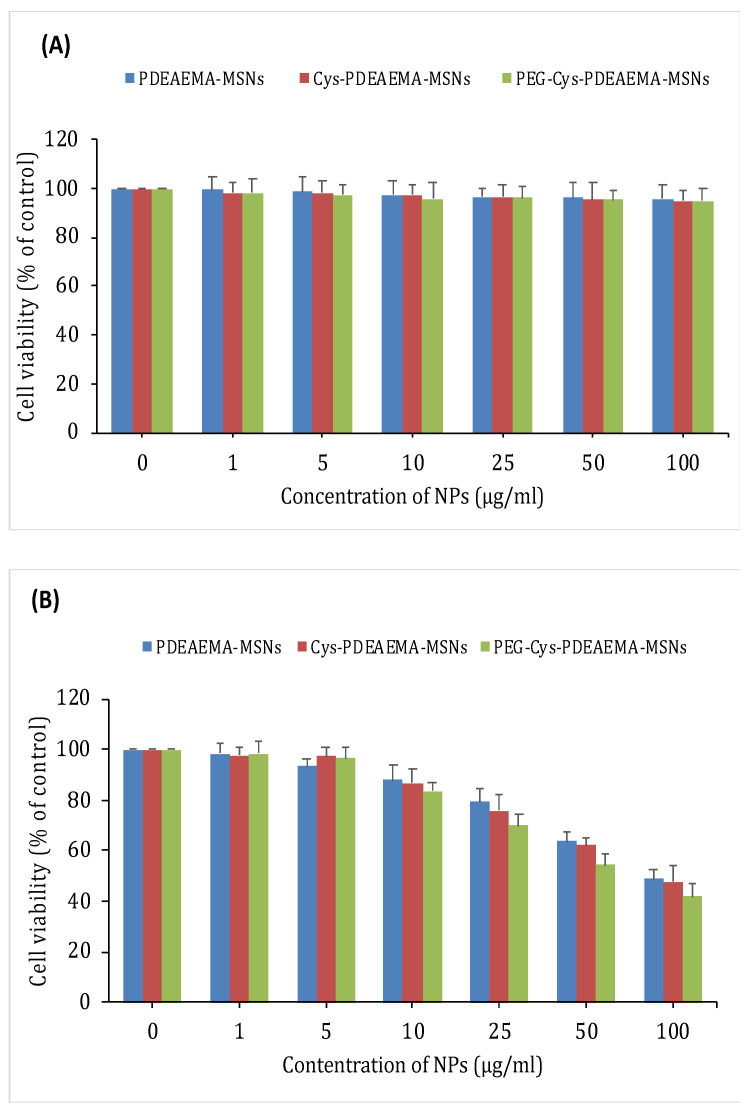
Cell viability of human liver cancer (HepG2) cells after exposure to MSN-PDEAEMA, MSN-PDEAEMA-Cys, and MSN-PDEAEMA-Cys-POEGMEMA. Cells were exposed for 24 h to different concentrations (1–100 µg/mL) of these materials and cell viability was measured through MTT assay. (**A**) PDEAEMA-MSNs, MSN-PDEAEMA-Cys, and MSN-PDEAEMA-Cys- POEGMEMA without drugs and (**B**) PDEAEMA-MSNs, MSN-PDEAEMA-Cys, and MSN-PDEAEMA-Cys-POEGMEMA with drugs.

The cytotoxicity/biocompatibility of the prepared nano-scale materials was determined in human liver cancer (HePG2) cells by MTT assay. Cells were exposed for 24 h to different concentrations (1–100 µg/mL) of MSN-PDEAEMA, MSN-PDEAEMA-Cys, and MSN-PDEAEMA-Cys-POEGMEMA-Cys. Figure 7A demonstrates that these different nanosystems did not induce cytotoxicity to HepG2 cells. These results suggest that the prepared nanocarriers have good biocompatibility and can be utilized as drug nanocarriers. Upon loading of DOX on these nanomaterials, they showed significant toxicity to human liver cancer cells (Figure 7B). These results suggest that the prepared nano-structured materials show good biocompatibility and can serve as nanocarriers for the delivery of anti-cancer drugs.

## 4. Conclusions

In summary, MSNs were successfully modified by PDEAEMA via SI-ARGET-ATRP, followed by the sequential addition of cysteine and POEGMEMA at the end of polymeric chains. Such nanomaterials were characterized by different techniques including DLS, surface zeta potential, XPS, FTIR, SEM, and TEM. The three designed nanocarriers exhibited a similar drug encapsulation efficiency of 69%. The drug release profiles for all nanosystems showed that the polymer swelling at low pH greatly accelerated the drug diffusion into aqueous solution. Furthermore, the anti-cancer activity of DOX-loaded MSN-PDEAEMA, MSN-PDEAEMA-Cys, and MSN-PDEAEMA-POEGMEMA was investigated. The in vitro cytotoxicity test showed that all three nanosystems exhibited excellent biocompatible properties. MSN-PDEAEMA-POEGMEMA loaded with DOX showed a stronger ability to kill the cancer cells compared to MSN-PDEAEMA and MSN-PDEAEMA-Cys and could be a potential effective agent in the treatment of cancer.

## Data Availability

The data presented in this study is openly available.

## Supplementary Materials

The following are available online at https://www.mdpi.com/2073-4360/13/5/823/s1, Figure S1: FTIR spectra of DOX loaded MSN-PDEAEMA, Figure S2: The average particle size of DOX loaded in MSN–PDEAEMA at different pH levels and the surface zeta potential DOX loaded in MSN-PDEAEMA.

## References

[1] World Health Organization (2020). Estimated Age-Standardized Incidence Rates (World) in 2020, All cancers, Both Sexes, All Ages. https://www.uicc.org/news/globocan-2020-new-global-cancer-data.

[2] Li Q., Sun A., Si Y., Chen M., Wu L. (2017). One-Pot Synthesis of Polysaccharide-Diphenylalanine Ensemble with Gold Nanoparticles and Dye for Highly Efficient Detection of Glutathione. Chem. Mater..

[3] Fleige E., Quadir M.A., Haag R. (2012). Stimuli-responsive polymeric nanocarriers for the controlled transport of active compounds: Concepts and applications. Adv. Drug Deliv. Rev..

[4] Khan R.U., Yu H., Wang L., Zhang Q., Xiong W., Zain-ul-Abdin W., Nazir A., Fahad S., Chen X., Elsharaarani T. (2020). Synthesis of polyorganophosphazenes and preparation of their polymersomes for reductive/acidic dual-responsive anticancer drugs release. J. Mater. Sci..

[5] Yi P., Wang Y., Zhang S., Zhan Y., Zhang Y., Sun Z., Li Y., He P. (2017). Stimulative nanogels with enhanced thermosensitivity for therapeutic delivery via β-cyclodextrin-induced formation of inclusion complexes. Carbohydr. Polym..

[6] Bae Y.H., Park K. (2020). Advanced drug delivery 2020 and beyond: Perspectives on the future. Adv. Drug Deliv. Rev..

[7] Barua S., Mitragotri S. (2014). Challenges associated with penetration of nanoparticles across cell and tissue barriers: A review of current status and future prospects. Nano Today.

[8] Lammers T., Kiessling F., Hennink W.E., Storm G. (2012). Drug targeting to tumors: Principles, pitfalls and (pre-) clinical progress. J. Control. Release.

[9] Bin H., Xin S., Bing Y., Song W., Youqing S., Hailin C. (2021). Recent advances in drug delivery systems for enhancing drug penetration into tumors. Drug Deliv..

[10] Sanadgol N., Wackerlig J. (2020). Developments of Smart Drug-Delivery Systems Based on Magnetic Molecularly Imprinted Polymers for Targeted Cancer Therapy: A Short Review. Pharmaceutics.

[11] Descalzo A.B., Martínez-Máñez R., Sancenón F., Hoffmann K., Rurack K. (2006). The Supramolecular Chemistry of Organic–Inorganic Hybrid Materials. Angew. Chem. Int. Ed..

[12] He Q., Zhang J., Shi J., Zhu Z., Zhang L., Bu W., Guo L., Chen Y. (2010). The effect of PEGylation of mesoporous silica nanoparticles on nonspecific binding of serum proteins and cellular responses. Biomaterials.

[13] Giri S., Trewyn B.G., Lin V.S. (2007). Mesoporous silica nanomaterial-based biotechnological and biomedical delivery systems. Nanomedicine.

[14] Slowing I.I., Trewyn B.G., Giri S., Lin V.S.Y. (2007). Mesoporous Silica Nanoparticles for Drug Delivery and Biosensing Applications. Adv. Funct. Mater..

[15] Carvalho G.C., Sábio R.M., Ribeiro T.C., Monteiro A.S., Pereira D.V., Ribeiro S.J.L., Chorilli M. (2020). Highlights in Mesoporous Silica Nanoparticles as a Multifunctional Controlled Drug Delivery Nanoplatform for Infectious Diseases Treatment. Pharm. Res..

[16] Wang X., Li X., Ito A., Yoshiyuki K., Sogo Y., Watanabe Y., Yamazaki A., Ohno T., Tsuji N.M. (2016). Silica Nanospheres: Hollow Structure Improved Anti-Cancer Immunity of Mesoporous Silica Nanospheres In Vivo. Small.

[17] Song N., Yang Y.-W. (2015). Molecular and supramolecular switches on mesoporous silica nanoparticles. Chem. Soc. Rev..

[18] Silveira C.P., Apolinário L.M., Fávaro W.J., Paula A.J., Durán N. (2016). Doxorubicin-Functionalized Silica Nanoparticles Incorporated into a Thermoreversible Hydrogel and Intraperitoneally Administered Result in High Prostate Antitumor Activity and Reduced Cardiotoxicity of Doxorubicin. ACS Biomater. Sci. Eng..

[19] Pandele A.M., Andronescu C., Ghebaur A., Garea S.A., Iovu H. (2019). New Biocompatible Mesoporous Silica/Polysaccharide Hybrid Materials as Possible Drug Delivery Systems. Materials.

[20] Keshavarz H., Khavandi A., Alamolhoda S., Naimi-Jamal M.R. (2020). pH-Sensitive magnetite mesoporous silica nanocomposites for controlled drug delivery and hyperthermia. RSC Adv..

[21] Nie D., Dai Z., Li J., Yang Y., Xi Z., Wang J., Li Y., Yu M., Zhang X., Shi X. (2020). Cancer-Cell-Membrane-Coated Nanoparticles with a Yolk-Shell Structure Augment Cancer Chemotherapy. Nano Lett..

[22] Beagan A., Lahmadi S., Alghamdi A., Halwani M., Almeataq M., Alhazaa A., Alotaibi K., Alswieleh A. (2020). Glucosamine Modified the Surface of pH-Responsive Poly(2-(diethylamino)ethyl Methacrylate) Brushes Grafted on Hollow Mesoporous Silica Nanoparticles as Smart Nanocarrier. Polymers.

[23] Alswieleh A.M., Alshahrani M.M., Alzahrani K.E., Alghamdi H.S., Niazy A.A., Alsilme A.S., Beagan A.M., Alsheheri B.M., Alghamdi A.A., Almeataqet M.S. (2019). Surface modification of pH-responsive poly(2-(tert-butylamino)ethyl methacrylate) brushes grafted on mesoporous silica nanoparticles. Des. Monomers Polym..

[24] Vallet-Regí M., Balas F., Arcos D. (2007). Mesoporous Materials for Drug Delivery. Angew. Chem. Int. Ed..

[25] Han J., Zhao D., Li D., Wang X., Jin Z., Zhao K. (2018). Polymer-Based Nanomaterials and Applications for Vaccines and Drugs. Polymers.

[26] Ribeiro A.M., Amaral C., Veiga F., Figueiras A. (2017). Chapter 8. Polymeric micelles as a versatile tool in oral chemotherapy. Design and Development of New Nanocarriers.

[27] Nabar G., Mahajan K., Calhoun M., Duong A., Souva M., Xu J., Czeisler C., Puduvalli V.K., Otero J.J., Wyslouzilet B.E. (2018). Micelle-templated, poly(lactic-*co*-glycolic acid) nanoparticles for hydrophobic drug delivery. Int. J. Nanomed..

[28] Rață D.M., Cadinoiu A.N., Atanase L.I., Bacaita S.E., Mihalache C., Daraba O.M., Gherghel D., Popa M. (2019). “In vitro” behaviour of aptamer-functionalized polymeric nanocapsules loaded with 5-fluorouracil for targeted therapy. Mater. Sci. Eng. C.

[29] Chen M., Hu J., Wang L., Li Y., Zhu C., Chen C., Shi M., Ju Z., Cao X., Zhang Z. (2020). Targeted and redox-responsive drug delivery systems based on carbonic anhydrase IX-decorated mesoporous silica nanoparticles for cancer therapy. Sci. Rep..

[30] Iurciuc-Tincu C.-E., Cretan M.S., Purcar V., Popa M., Daraba O.M., Atanase L.I., Ochiuz L. (2020). Drug Delivery System Based on pH-Sensitive Biocompatible Poly(2-vinyl pyridine)-b-poly(ethylene oxide) Nanomicelles Loaded with Curcumin and 5-Fluorouracil. Polymers.

[31] Duan R., Xia F., Jiang L. (2013). Constructing Tunable Nanopores and Their Application in Drug Delivery. ACS Nano.

[32] Khorsand B., Lapointe G., Brett C., Oh J.K. (2013). Intracellular Drug Delivery Nanocarriers of Glutathione-Responsive Degradable Block Copolymers Having Pendant Disulfide Linkages. Biomacromolecules.

[33] Mo R., Jiang T., DiSanto R., Tai W., Gu Z. (2019). ATP-triggered anticancer drug delivery. Nat. Commun..

[34] Mura S., Nicolas J., Couvreur P. (2013). Stimuli-responsive nanocarriers for drug delivery. Nature Mater..

[35] Vaupel P., Kallinowski F., Okunieff P. (1989). Blood flow, oxygen and nutrient supply, and metabolic microenvironment of human tumors: A review. Cancer Res..

[36] Alswieleh A.M., Beagan A.M., Alsheheri B.M., Alotaibi K.M., Alharthi M.D., Almeataq M.S. (2020). Hybrid Mesoporous Silica Nanoparticles Grafted with 2-(tert-butylamino)ethyl Methacrylate-b-poly(ethylene Glycol) Methyl Ether Methacrylate Diblock Brushes as Drug Nanocarrier. Molecules.

[37] Beagan A.M., Alghamdi A.A., Lahmadi S.S., Halwani M.A., Almeataq M.S., Alhazaa A.N., Alotaibi K.M., Alswieleh A.M. (2021). Folic Acid-Terminated Poly(2-Diethyl Amino Ethyl Methacrylate) Brush-Gated Magnetic Mesoporous Nanoparticles as a Smart Drug Delivery System. Polymers.

[38] Mishra P., Nayak B., Dey R.K. (2016). PEGylation in anti-cancer therapy: An overview. Asian J. Pharm. Sci..

[39] Sato Y., Hatakeyama H., Sakurai Y., Hyodo M., Akita H., Harashima H. (2012). A pH-sensitive cationic lipid facilitates the delivery of liposomal siRNA and gene silencing activity in vitro and in vivo. J. Control. Release.

[40] Yang C., Xiao J., Xiao W., Lin W., Chen J., Chen Q., Zhang L., Zhang C., Guo J. (2017). Fabrication of PDEAEMA-based pH-responsive mixed micelles for application in controlled doxorubicin release. RSC Adv..

[41] Feng J., Wen W., Jia Y.-G., Liu S., Guo A.J. (2019). pH-Responsive Micelles Assembled by Three-Armed Degradable Block Copolymers with a Cholic Acid Core for Drug Controlled-Release. Polymers.

[42] Kang N., Perron M.-È., Prud’homme R.E., Zhang Y., Gaucher G., Leroux J.-C. (2005). Stereocomplex Block Copolymer Micelles: Core−Shell Nanostructures with Enhanced Stability. Nano Lett..

[43] Nik A.B., Zare H., Razavi S., Mohammadi H., Ahmadi P.T., Yazdani N., Bayandorif M., Rabiee N., Mobarakeh J.I. (2020). Smart drug delivery: Capping strategies for mesoporous silica nanoparticles. Microporous Mesoporous Mater..

[44] Atanase L.I. (2021). Micellar Drug Delivery Systems Based on Natural Biopolymers. Polymers.

[45] Bilalis P., Tziveleka L.-A., Varlas S., Iatrou H. (2016). pH-Sensitive nanogates based on poly(l-histidine) for controlled drug release from mesoporous silica nanoparticles. Polym. Chem..

[46] Mosmann T. (1983). Rapid colorimetric assay for cellular growth and survival: Application to proliferation and cytotoxicity assays. J. Immunol. Methods.

[47] Ahamed M., Akhtar M.J., Siddiqui M.A., Ahmad J., Musarrat J., Al-Khedhairy A.A., AlSalhi M.S., Alrokayan S.A. (2011). Oxidative stress mediated apoptosis induced by nickel ferrite nanoparticles in cultured A549 cells. Toxicology.

[48] Alenazi N., Hussein M., Alamry K., Asiri A. (2018). Nanocomposite-Based Aminated Polyethersulfone and Carboxylate Activated Carbon for Environmental Application. A Real Sample Analysis. C J. Carbon Res..

[49] Mohammed I.A., Mustapha A. (2010). Synthesis of New Azo Compounds Based on *N*-(4-Hydroxypheneyl)maleimide and *N*-(4-Methylpheneyl)maleimide. Molecules.

