# Supplementary material for: Poly(oligo(ethylene glycol) methyl ether methacrylate) Capped pH-Responsive Poly(2-(diethylamino)ethyl methacrylate) Brushes Grafted on Mesoporous Silica Nanoparticles as Nanocarrier
Source: Polymers (Basel). 2021 Mar 8;13(5):823. doi: 10.3390/polym13050823 (PMC7962535; doi:10.3390/polym13050823)
Supplement: Supplementary file 1 [file polymers-13-00823-s001.pdf]

Poly(oligo(ethylene glycol) methyl ether methacrylate) capped pH-responsive poly(2-(diethylamino)ethyl methacrylate) brushes grafted on mesoporous silica nanoparticles as nanocarrier

Khalid M. Alotaibi,[1,4,‡] Abdurrahman A. Almethen,[2,*] Abeer M. Beagan,[1,‡] Latifah H. Alfhaid,[3] Maqusood Ahamed,[4] Ahmed M. El-Toni,[4] and Abdullah M. Alswieleh [1,*]

[1] Department of Chemistry, College of Science, King Saud University, Riyadh, Kingdom of Saudi Arabia.

[2] King Abdulaziz City for Science and Technology, Riyadh, Saudi Arabia.

[3] Department of Physics, College of Science, University of Ha'il, Ha'il, Kingdom of Saudi Arabia

[4] King Abdullah Institute for Nanotechnology, King Saud University, Riyadh, Saudi Arabia.

* Correspondence: aswieleh@ksu.edu.sa (A.M.A); amethen@kacst.edu.sa (A.A.A)

† These authors contributed equally.

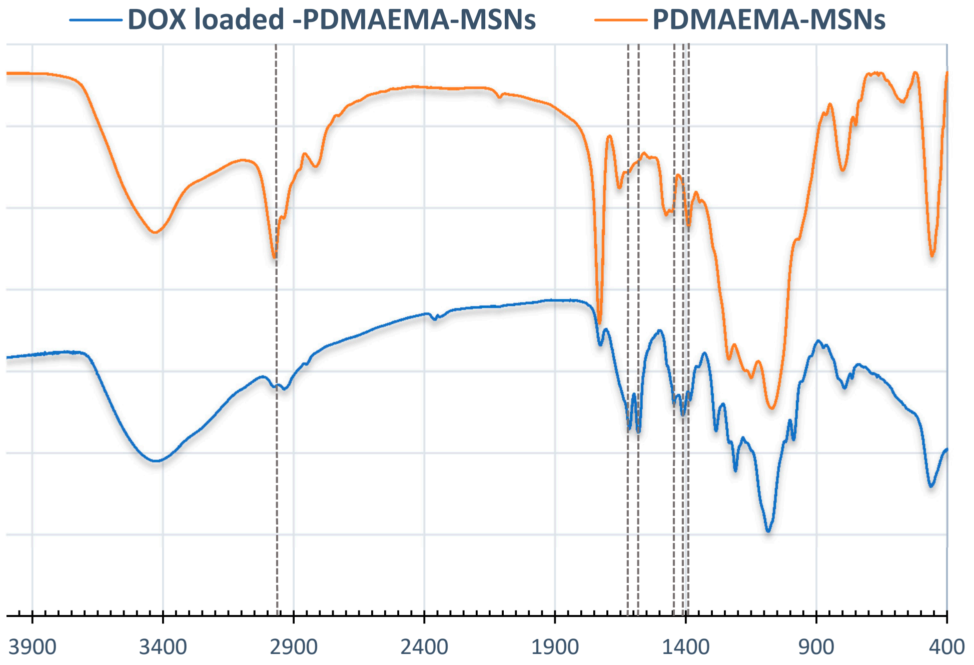

Figure S1: FT-IR spectra of MSN-PDEAEMA and DOX loaded MSN-PDEAEMA.

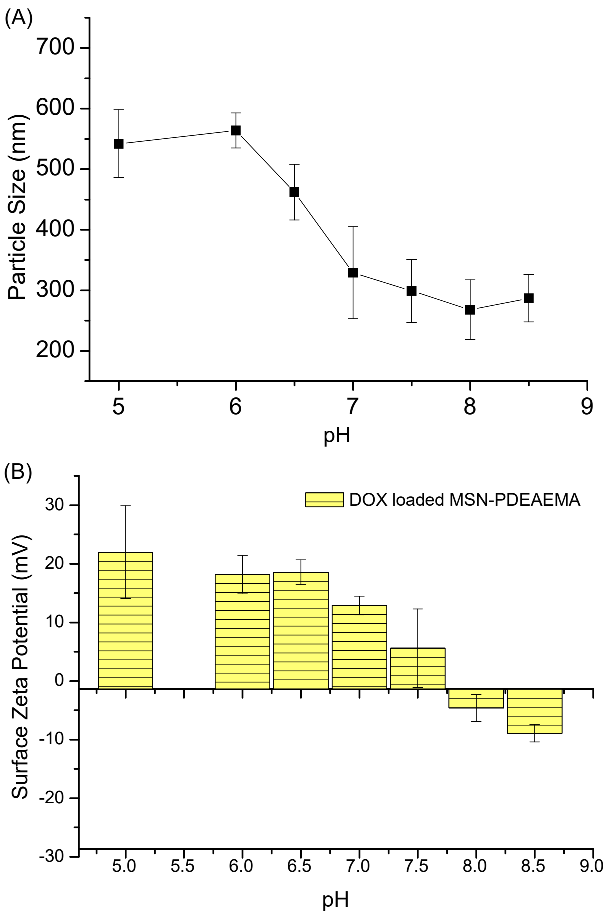

Figure S2: (A) The average particle size of DOX loaded MSN-PDEAEMA. (B) The surface zeta potential of DOX loaded MSN-PDEAEMA.
